# A Case of Imperforate Rectum in Which Lumbar Colotomy Was Performed

**Published:** 1884-12-20

**Authors:** John H. Packard

**Affiliations:** Surgeon to the Pennsylvania Hospital and to St. Joseph’s Hospital, Philadelphia


### A CASE OF IMPERFORATE RECTUM IN WHICH LUMBAR COLOTOMY WAS PERFORMED.

By JOHN H. PACKARD, M. D.,
Surgeon to the Pennsylvania Hospital and to St. Joseph's Hospital, Philadelphia.

In the last number of this journal there was presented by Dr. W. H. Haynes a very interesting report of a case of imperforate rectum, in which left inguinal colotomy was practiced, the child living for over eleven months. In the instance which I desire now to place upon record the colon was opened in the left loin, and the child's life was prolonged nineteen weeks and a few days. I trust it will be thought to possess sufficient interest and instruction to warrant me in publishing it.

The case occurred in the practice of Dr. C. R. Prall, the child being born of parents in comfortable circumstances, on the 22d of

March, 1884. At Dr. Prall's request I saw it on the 23d, when it was thirty-six hours old. It was a well-developed male, but had been from the time of birth in a state of great and increasing discomfort, fretting and crying incessantly, with almost constant vomiting.

On examination, there was found at the normal position of the anus an opening, which led into a cul-de-sack. Into this the little finger could be readily passed to the distance of about an inch. A probe could be carried upward, through a very narrow continuation of this passage, about a quarter of an inch further. Nothing could be felt indicating the position of the terminal portion of the bowel above; nor did the lower opening of the cul-de-sac give the impression of a regularly-formed sphincter.

It seemed to us that the course most likely to save the child's life, and involving the least risk, would be the formation of an artificial anus in the left loin; and we hoped that the natural passage might subsequently be established upon indications furnished by the insertion of instruments through the orifice so made. The parents, the matter being fully explained to them, were anxious to have anything done to save the child's life, although they were made aware that the artificial anus might have to remain as a permanence.

No difficulty was encountered in the operative procedure, except that arising from the small size of the parts. An incision was made downward and outward from the upper and outer angle of the quadratus lumborum muscle, nearly to the crista ilii, about an inch and a half; the tissues were then divided on a grooved director, until the colon was found lying just across the middle of the wound. The little tube was so slender, and its wall so thin, that it seemed as if it could hardly be manipulated without rupture. It was, however, secured by two ligatures of fine raw silk, and an opening made between them, giving exit to a very large quantity of meconium. The edges were then secured by sutures to the skin, which itself seemed hardly equal to the slightest strain. A bit of lint spread with carbolized cerate was applied, and over this a sheet of absorbent cotton, the whole being kept in place by the flannel binder.

The child became immediately tranquil, ceased to vomit, and nursed well. Within a few hours of the operation very copious oozing of blood began from the wound, and continued for a number of hours, until Dr. Prall applied some tannic acid upon cotton, when it ceased, and did not recur. Rapid healing ensued, and, in fact, the tendency was to excessive contraction of the orifice; so that frequent doses of castor oil had to be given, and every day or two instrumental dilatation was required. I made careful efforts at enlarging the opening with a button-pointed bistoury, but the tendency to closure was very strong, and had to be constantly counteracted. I made an instrument like a wire speculum, using a hair-pin for the purpose, and giving it a pretty strong outward spring; but finally resorted to tents made of "marine lint," with better success.

A few days after the operation, and at different times subsequently, we passed a very long and slender leaden probe, with a heavy plummet-shaped end, in the hope that the latter would gravitate into the blind extremity of the gut below, and indicate a favorable spot for opening it. But neither with this, nor with other instruments which we tried, could we get any guidance such as we sought.

The child's general condition continued very favorable for some time, but toward the latter part of July he began to lose flesh and show signs of weakness. In spite of abundant stimulation and nourishment, the failure of strength became more and more evident, and he died on the 7th of August, one hundred and thirty-seven days from the time of the operation.

An autopsy made the same day showed very great distension of the colon with semi-liquid and perfectly healthy fecal matter. The sigmoid flexure was extremely enlarged, and the bowel strongly adherent to the wall of the abdomen at the point where the artificial opening had been made. The gut ended in a wide cul-de-sac, which, although close to that mentioned as receiving the point of the finger, was not close enough to afford a guide for incision by bulging against it. No fibrous cord or other connection could be detected between the two.

On comparing the case now detailed with that reported by Dr. Haynes, it will be seen that the object aimed at was the same in both, viz, to open the bowel with a view not only to immediate relief, but to the subsequent establishment of the natural passage and closure of that artificially made. I freely admit that inguinal colotomy, chosen by Dr. Haynes with this view, and successful in its result, was the better course, and I should adopt it myself in any similar case in future. My preference for the operation in the loin was based upon a belief in its greater safety, as well as upon my familiarity with it as practised upon the adult. But the risk involved in opening the peritoneal cavity, as well as the somewhat greater difficulty of the operation, would seem to be outweighed by the advantage of the readier access to the cul-de-sac forming the terminal part of the gut, and by the better prospect of thus remedying the abnormal condition.

One point in my own case seems to me especially instructive, and that is the strong tendency to closure manifested by the artificial opening. Hoping to succeed in establishing the natural passage, I was very guarded in my interference with the wall of the gut. Indeed, my object was, after affording an exit to the meconium, to have simply room for the introduction of instruments. In the adult, if the orifice is larger, the mucous membrane is apt to pouch out very freely, like the anus of the horse in defecation, and I have seen much annoyance result. But I believe that it would have been better, as regards the mere saving of the child's life, if I had made an ample opening into the gut, so as to allow of free evacuation of the feces.

It is very satisfactory to be able to record a triumph of surgery; but sometimes the report of a failure is of even greater value, if it serves to mark the channel by which success may be reached.—*Am. Jour. Med. Science.*

